# The therapeutic potential of matcha tea: A critical review on human and animal studies

**DOI:** 10.1016/j.crfs.2022.11.015

**Published:** 2022-11-23

**Authors:** Sara Sokary, Maha Al-Asmakh, Zain Zakaria, Hiba Bawadi

**Affiliations:** a Department of Human Nutrition, College of Health Sciences, QU-Health, Qatar University, Doha, 2713, Qatar; b Department of Biomedical Sciences, College of Health Sciences, QU-Health, Qatar University, Doha, 2713, Qatar; c Biomedical Research Center, Qatar University, Doha, 2713, Qatar

**Keywords:** Matcha tea, Catechins, Cognitive function, Cardio-metabolic, Anti-tumor

## Abstract

Matcha is a powdered form of Japanese green tea that has been gaining global popularity recently. Matcha tea has various health benefits, including an enhancing effect on cognitive function, cardio-metabolic health, and anti-tumorogenesis. To date, randomized clinical trials (RCT) showed that matcha decreases stress, slightly enhances attention and memory, and has no effect on mood. Results regarding the effect of matcha on cognitive function are contradictory and more RCTs are warranted. The cardio-metabolic effects of matcha have only been studied in animals, but findings were more homogenous. Consuming matcha with a high-fat diet resulted in decreased weight gain velocity, food intake, improved serum glucose and lipid profile, reduced inflammatory cytokines and ameliorated oxidative stress. Evidence regarding the anti-tumor function of matcha is very limited. Findings showed that matcha can affect proliferation, viability, antioxidant response, and cell cycle regulation of breast cancer cells. Nonetheless, more studies are needed to examine this effect on different types of cancer cells, and there is also a need to verify it using animal models. Overall, the evidence regarding the effect of matcha tea on cognitive function, cardio-metabolic function, and anti-tumor role is still limited, and conclusions cannot be drawn.

## Highlights

• Matcha decreases stress and anxiety, improves memory and short- and long-term cognitive function in humans, while having no effect on mood.
• Studies on mice showed that matcha enhanced spatial learning, upregulated Brain-Derived Neurotrophic Factor and insulin-degrading enzyme leading to reduced neuroinflammation.
• Matcha improves lipid profile and glucose metabolism, reduces inflammation and results in lower weight gain due to high fat diet in mice.
• In vitro studies showed the anti-tumor effect of matcha by reducing viability, mitochondrial metabolism, ATP production and oxygen consumption of breast cancer cells by disrupting cell cycle regulation and interleukin signaling.

## Graphical abstract

**Figure:**
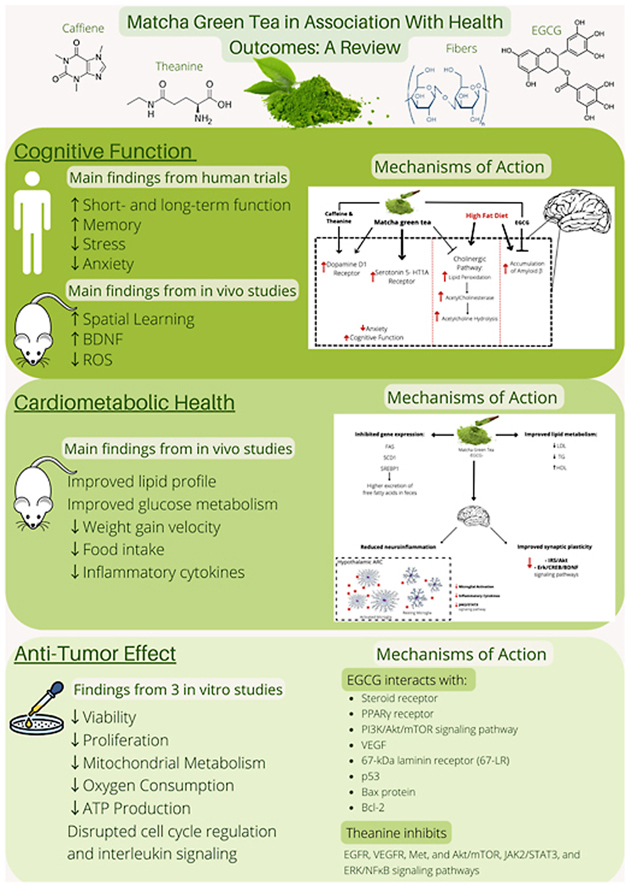

## Abbreviations

EGCG: Epigallocatechin-3-gallate
BDNF: Brain-derived neurotrophic factor
Aβ: Amyloid beta
HFD: High-fat diet

## 1 Introduction

Tea is one of the most commonly consumed beverages worldwide, second only to water (Wolf et al., 2008). Matcha tea is a powdered form of Japanese green tea (*Camellia sinensis*) (Horie et al., 2017) used in the traditional tea ceremony and in various food products in Japan, and it has been gaining global popularity recently. Matcha comes in a powdered form, hence, the leaf is consumed completely, while in other types of tea that come in loose leaf form, the extraction of the soaked leaves is consumed. The traditional way to grow Japanese green tea is by covering the tea bushes using bamboo mats to shade the leaves from direct sunlight for the majority of the growth period (Farooq and Sehgal, 2018). After that, only the high-grade young tea leaves are selected and immediately steamed briefly to prevent their oxidation. Then their stems, veins and impurities are removed (Farooq and Sehgal, 2018). This process allows the plant to produce higher amounts of amino acids and bioactive compounds like chlorophyll and theanine, giving matcha tea its characteristic vibrant green color and non-bitter taste (Wolf et al., 2008). The leaves are then ground with a ceramic mill to produce a fine powder, that is whisked with water at a relatively low temperature (70–80 °C) to make a creamy and frothy beverage (Kaneko et al., 2006). The taste profile for matcha is unique, with a rich and complex umami, fresh green, roast, and vegetable-like taste sensations (Kaneko et al., 2006).

## 2 Chemical composition of match tea

The nutrients in matcha tea are 60–70% insoluble ingredients such as fat-soluble vitamins, insoluble dietary fibers, chlorophylls, and proteins. While the soluble ingredients constitute 30–40% which includes polyphenols, water-soluble vitamins, caffeine, water-soluble dietary fibers, amino acids, saponin, and minerals (Maeda-Yamamoto et al., 2013). Given their unique farming and harvesting processes, the concentrations of bioactive compounds are higher in matcha tea than in other types of green tea. As matcha tea leaves are protected from sunlight before harvesting, it contains lower catechin content than other types of green tea prepared from leaves grown in sunlight (Goto et al., 1996; Ikegaya et al., 1984). However, once matcha is dissolved in water, it produces 3 times more catechins than the loose-leaf form of green tea (Fujioka et al., 2016). Furthermore, caffeine content in matcha is higher because the buds and young leaves of tea plants contain more caffeine than mature leaves (Ashihara and Suzuki, 2004). The balance between all these components, e.g., theanine, catechins, caffeine, define the quality of the green tea. Matcha also has a high level of “umami” flavor profile due to its high content of amino acids (Ruan et al., 2010). Consequently, matcha is considered a high-grade green tea due to its high content of amino acids and caffeine and low content of catechins in contrast to other popular types of green tea (see Table 1).

**Table 1:** Summary of the chemical composition of matcha tea from different studies.

	(Iwai et al., 2021) (mg/100g)	(Baba et al., 2021a) (mg/2070 mg)	(Li et al., 2021) (mg/g DW)		(Xu et al., 2016) (%)	(Zhou et al., 2021) (%)
Matcha Tea Type	Kyoeiseicha, standard matcha-grade leaves	Hojin no shiro (ceremonial grade matcha)	Seven-Star Matcha Tea	Selenium-Enriched Matcha Tea	N/A	N/A
Components						
Total Polyphenols	–	170	–	–	7.7	19.84 ± 0.67
*Epigallocatechin gallate*	5900	105.6	13.462 ± 0.206	24.264 ±0.27	1.46	7.02 ± 0.93
*Epigallocatechin*	1900	33.1	20.865 ± 0.073	9.425 ± 0.05	1.1	1.15 ± 0.09
*Epicatechin gallate*	890	20.3	8.727 ±0.079	9.378 ± 0.038	0.4	0.83 ± 0.13
*Epicatechin*	360	8.1	7.421 ± 0.133	3.379 ± 0.052	0.49	0.44 ± 0.02
*Gallocatechin*	120	1.6	5.736 ± 0.075	7.409 ± 0.091	–	1.15 ± 0.1
*Gallocatechin gallate*	67	1.4	–	9.03 ± 0.069	0.3	1.27 ± 0.2
*Catechin*	35	0.8	–	–	1.30	0.08 ± 0.11
*Catechin gallate*	4.9	0.1	0.375	4.844 ± 0.109	0.09	0.09 ±
Caffeine	–	66.2	28.715 ± 0.158	29.34 ± 0.27	–	6.58 ± 0.01
Theanine	1800	48	–	–	–	–
Total chlorophyll	996	–	–	–	–	–
Amino acids	–	–	–	–	2.52	4.19 ±0.01
Sugars	–	–	–	–	5.54	3.14 ± 0.15
Fiber	–	–	–	–	38.5	–

— for values not measured.

## 3 Health benefits of matcha

The health benefits associated with Japanese green tea have been linked to its content of natural antioxidants (Kurleto et al., 2013), such as polyphenols: various compounds that account for as much as 30% of matcha tea's dry weight (Mandel et al., 2005; Komes et al., 2010). Polyphenols are powerful antioxidants, almost as effective as vitamins like vitamin C, vitamin E, carotene, and tocopherol. In matcha, catechins make up 90% of these polyphenols (Tachibana 2009). The main four types of catechins are (−)-epicatechin (EC), (−)-epicatechin-3-gallate (ECG), (−)-epigallocatechin (EGC) and (−)-epigallocatechin-3-gallate (EGCG), where EGCG is the most abundant and active (Kochman et al., 2020). Multiple studies compared matcha, the powder form of green tea, to the traditional loose-leaf form of green tea, and results suggested that matcha might function differently from loose-leaf tea due to its higher content of catechins and residues (Fujioka et al., 2016; Zhou et al., 2021). Another study compared the effect of the extract, residues, and entire matcha, and found that the residue contains mostly water-insoluble fibers and contributes greatly to the health-promoting benefits of matcha (Xu et al., 2016). Specific components of matcha such as catechins, caffeine and theanine are well studied and have been associated with multiple health-promoting effects. A recent meta-analysis concluded that caffeine may promote weight and fat loss, facilitating BMI reduction (Tabrizi et al., 2019). Another meta-analysis of RCTs by (Zheng et al., 2013) showed that catechins from green tea significantly lowered fasting blood glucose concentrations (−1.48 mg/dL; 95% CI: −2.57, −0.40 mg/dL) but did not affect fasting blood insulin, HbA1c, or HOMA-IR levels. Caffeine ingestion was also linked to enhanced muscle strength and power, specifically in the upper body muscles (Grgic et al., 2018). The combined effect of theanine and caffeine was systematically reviewed recently and showed that it results in clinically significant enhancements in cognitive function (Sohail et al., 2021). Similarly, theanine (Yoneda et al., 2019), caffeine (Fredholm et al., 1999, Smit and Rogers, 2000) and catechins (Baba et al., 2021a) were found to have an enhancing effect on cognitive function.

### 3.1 Matcha tea and the improvement in cognitive function

Matcha first gained popularity for the favorable changes it exerts on cognitive function, memory, and focus (see Table 2). Bioactive compounds abundant in matcha such as caffeine, theanine and catechins have been linked to multiple benefits to the cognitive function. Caffeine is a psychoactive stimulant that results in increased alertness and enhanced cognitive performance as reported by multiple studies and systematic reviews (Smit and Rogers, 2000; Snel and Lorist, 2011; McLellan et al., 2016; Panza et al., 2015). In addition, previous literature showed that theanine influences neurogenesis and cognitive function positively in a manner independent of other compounds found in matcha (Yoneda et al., 2019; Camfield et al., 2014). However, caffeine can strengthen the effect of theanine on neurophysiological functions like attention (Dietz and Dekker, 2017). Additionally, recent studies on EGCG, the main catechin in matcha, demonstrated that it has potential neuroprotective effects against neurological disorders by acting as an active compound that ameliorates cognitive defects (Pervin et al., 2019; Mi et al., 2017). The results from genetic study on mice suggested that catechins in matcha could slow age-related cognitive decline by increasing expression of genes involved in long-term changes in plasticity of synapses and neuronal circuits (Unno et al., 2020).

**Table 2:** Summary of studies investigating the effects of matcha tea on cognitive function.

Reference	Type	Year	Treatment and Dose	Duration of exposure	Outcome	Methods	Results
Human Studies							
(Sakurai et al. (2020)	RCT 54 subjects	2020	1.5 g of matcha twice a day as capsule vs placebo	12 weeks	Cognitive memory function and impulsivity	Montreal Cognitive Assessment (MoCA) score, Mini-Mental State Examination (MMSE), Wechsler Memory Scale—Delayed Recall (WMS-DR), Barratt Impulsiveness Scale-11 (BIS-11)	Language domain of the MoCA score. (effect seen only in women)
							**X** No Effect in cognitive function
Dietz et al. (2017)	RCT in 23 subjects	2017	4.0 g matcha per test product	60 min	Cognitive function and mood	Cognitive Drug Research battery (CDR)	**X** No effect on mood.
						30-item short form of the POMS as a subjective mood measure	Slight effect on speed of attention and working memory.
Baba et al. (2021b)	RCT in 42 subjects	2021	2 g of matcha per day	2 weeks	Attentional function	Cognitraxe test	Reaction time
						Uchida–Kraepelin test (UKT)	Cognitive function
						Visual Analogue Scale (VAS) to assess subjective fatigue	X No effect on fatigue, concentration, thinking ability, and energy level
Unno et al. (2018)	RCT in 39 students		3 g matcha tea bags	15 days	Anxiety and physiological stress	State-trait anxiety inventory (STAI) test	Salivary amylase
						Subjective stress was evaluated using visual analogue scales (VAS)	Subjective stress
						Morning and evening salivary amylase to assess physiological stress	
Baba et al. (2021a)	RCT in 51 subjects	2021	Matcha capsules vs caffeine capsules vs placebo	Single dose or for 12 consecutive weeks	Cognitive Function following mild, acute psychological stress	Cognitrax	Caffeine:
			9 capsules daily manufactured by Pharmaceutical companies			UKT results	Extension of reaction time of cognitive function tasks
						Japanese version of Mini-Mental State Examination (MMSE-J)	Matcha:
						serum Aβ, Aβ, sAPPα, APP770, and BDNF levels.	Enhanced performance under stress (better than caffeine)
Animal Studies							
Unno et al. (2018)	Male ddY Mice	2018	0, 10, 17, 33, 50, and 100 mg/kg body weight of matcha with normal diet	7 days	Stress, evaluated by suppression of adrenal hypertrophy	Weight of adrenal gland	Adrenal gland weight
Iwai et al. (2021)	Flt1-DsR mice	2021	Normal diet + 2% matcha (equivalent to 6.6–7.3 g)	30 weeks	Brain function through capillary aging	Capillary density	Cortical capillary density in the deep layer
						Aortic ring assay	Angiogenic potential
						Tube formation assay	Protects brain function by preventing vascular aging in the brain cortex
Kim et al. (2020)	Male C57BL/6 mice	2020	HFD + 20 mg vs 50 mg of matcha per kg of body weight vs HFD (control)	14 weeks	Cognitive impairment caused by hepatic and cerebral insulin resistance	OGTT	Spatial learning and memory function
						Intraperitoneal GTT	Improved tracked movements, long-term learning
						Y-Maze Test	weight gain velocity
						Passive Avoidance Test	suppressed hyperglycemic state
						Morris Water Maze Test	Accumulation of perirenal retroperitoneal, epididymal, and mesenteric fat
						Organ weight change	Improved hepatic mitochondria function
						GOT, GPT, LDH, TC, TG, HDL, LDL, HDL:LDL ratio	Upregulated BDNF and IDE
						Superoxide Dismutase, Reduced Glutathione, Malondialdehyde	
						Acetylcholine, Acetylcholinesterase.	
						Mitochondrial ROS Contents, Mitochondrial Membrane Potential	
Kim et al. (2021)	Male BALB/c mice	2021	20 mg or 40 mg per kg of body weight aqueous extract of matcha vs control	12 weeks	Particulate matter (PM)2.5-induced systemic inflammation	Y-Maze Test	Improved cholinergic system
						Passive Avoidance Test	Increased mitochondrial membrane potential (MMP) and ATP contents
			The mice were exposed to PM2.5 at a 500 μg/m3 concentration for 5 h per day			Morris Water Maze Test	Lower reactive oxygen species (ROS)
						plasma ferric reducing/antioxidant power (FRAP)	Improved spatial learning and memory function
						Superoxide Dismutase, Reduced Glutathione, Malondialdehyde	antioxidant system, cholinergic system, and mitochondrial activity
						Acetylcholine, Acetylcholinesterase.	
						Mitochondrial ROS Contents, Mitochondrial Membrane Potential, ATP Contents	

Abbreviations: Aβ, Amyloid beta; APP770, Amyloid β precursor protein 770; BDNF, Brain-derived neurotrophic factor; GOT, Glutamic oxaloacetic transaminase; GPT, glutamic pyruvic transaminase; HDL, high-density lipoprotein; IDE, Insulin-degrading enzyme; LDH, Lactate dehydrogenase; LDL, low-density lipoprotein; OGTT, Oral glucose tolerance test; RCT, Randomized clinical trial; sAPPα, Secreted amyloid β precursor protein-alpha; TC, Total cholesterol; TG, triglycerides.

#### 3.1.1 Match tea and cognitive function: human studies

In humans, the effect of matcha was studied in relation to its effect on memory, brain function, focus, among others. A recent randomized placebo-controlled study examined the effect of matcha tea intake on acute psychological stress (Baba et al., 2021b). Participants consumed 2.07 g of matcha every day, containing 50.3 mg of theanine, 171.0 mg of catechins, and 72.5 mg of caffeine. After two weeks, they evaluated the memory, attention, facial expression recognition, working memory, visual information, and motor function in the matcha and the placebo groups. Matcha intake caused a statistically significant reduction in reaction time and an increase in the number of hits in perception of emotions test, indicating an enhanced cognitive function. However, matcha did not cause a reduction in fatigue, nor an improvement in the concentration, thinking ability, or energy levels of the participants that consumed it (Baba et al., 2021b). The same research group conducted another study to compare the effect of matcha and caffeine on cognitive function after mild acute psychological stress (Baba et al., 2021a). Fifty-one participants consumed 9 capsules of matcha or caffeine only or placebo, and the effect was measured after the first capsule (single dose) and after continuous ingestion for 12 weeks (continuous intake). The results showed that caffeine improved attentional function after a single dose, explaining the decreased reaction time seen following a single dose of matcha. The effect of continuous intake of matcha lead to an enhanced performance under stress load compared to caffeine. No effect was seen on the dementia-related blood biomarkers and other test measures. Their results indicate that matcha has an anti-stress function with continuous intake which might lead to maintenance or improvement of attention, and that the effect of caffeine in accelerating reaction and focus is only acute (Baba et al., 2021a). Another RCT by Unno et al. (2018) examined the effect of matcha ingestion on anxiety and physiological stress, by assessing the subjective stress and salivary amylase, respectively. Thirty-nine students drank 3 g of matcha daily for 15 days, which lead to a significant decrease in anxiety measured by visual analogue scale and State-trait anxiety inventory (STAI) test and lowered physiological stress compared to placebo (Unno et al., 2018).

Furthermore, an RCT by Sakurai et al. studied the cognitive functions of clinically normal elderly people after supplementation with 3 g of matcha in a drink for 12 weeks (Sakurai et al., 2020). Only women showed an enhancement in the language domain after the trial, and an association between higher vitamin K consumption in deficient individuals and cognitive function. The authors explained their results through the higher resilience of healthy older women to age-related cognitive decline, as well as a possible enhanced effect of vitamin K on women's brain rather than in men. Notably, this study only included 15 male participants out of the 54 that completed the trial, which indicates a need for larger sample size to confirm this effect in males. It is also worth mentioning that all studies discussed so far were conducted in the Japanese population, known for their regular consumption of green tea, which means that such intervention could have not increased consumption in some participants, possibly affecting the observed results (Sakurai et al., 2020). One RCT conducted in Netherlands in 2017 studied the effect of 4 g of matcha on attention, information processing, working memory, and episodic memory 60 min after matcha consumption to test for the acute effects of matcha tea consumption (Dietz et al., 2017). Each participant consumed four test products: matcha tea, matcha tea bar, placebo tea, and placebo bar in a randomized manner. The study provided little evidence that matcha could affect cognitive performance in short-term, as mood was not affected, and speed of attention and working memory were only slightly affected. Furthermore, EGCG, for example, reaches peak plasma concentration after 1.3–2.4 h, which is twice the time between administration and testing in this study (Dietz et al., 2017). Available evidence points at an overall enhancing function both acutely and after long-term ingestion, however, the studies are heterogenous and small in number, therefore, it is hard to draw conclusions based on them.

#### 3.1.2 Match tea and cognitive function: animal studies

Multiple animal studies examined the effect of matcha tea on cognitive function. The *in vivo* arm of the study by Unno et al. (2018) tested the effect of 6 levels of matcha intake on mice and showed a dose-dependent reduction in adrenal gland weight, which indicates lower psychological stress, although matcha ingestion had no effect on food intake or body weight (Unno et al., 2018). A more recent study showed that matcha has the potential to prevent vascular aging by protecting the neuron density in layer 1 of the brain cortex, suggesting that matcha tea ingestion has a protective effect on brain function by preserving vascular health (Iwai et al., 2021). The study by Kim et al. (2020) examined the effect of matcha tea in reversing high-fat diet induced cognitive dysfunction (Kim et al., 2020). The study showed that matcha slowed weight gain, improved hyperglycemia, and lowered perirenal retroperitoneal, epididymal, and mesenteric fat accumulation. Matcha had a significant protective effect on cognitive function in term of memory, long-term learning, tracked movements, and spatial learning. Finally, matcha was able to also upregulate Brain-derived neurotrophic factor (BDNF) and insulin-degrading enzyme (IDE) compared to the placebo group. BDNF facilitates synaptic transmission and support long-term potentiation that is associated with learning and memory, while IDE is a principal regulator of Amyloid β levels in neuronal and microglial cells, and both molecules reduce neuroinflammation and protect cognitive function. The same research group conducted another study to examine the effect of matcha in mice exposed to air pollution by exposing them to particulate matter at a concentration ≤2.5 μM (Kim et al., 2021). Matcha was given as an aqueous extract at 20 or 40 mg/kg of body weight and was able to protect cognitive function by ameliorating systematic inflammation. The matcha group was able to protect against antioxidant deficit in pulmonary, dermal, and cerebral tissues, showed improved cholinergic system in the brain and prevented mitochondrial dysfunction. Mice on matcha also did better on the behavioral tests reflecting a maintained cognitive function (Kim et al., 2021). The overall evidence from animal studies points at an enhancing effect of matcha on cognitive function, but available studies are limited, and more studies are needed to be able to draw conclusions about the effect of matcha on cognitive function in animal models.

#### 3.1.3 Plausible mechanisms matcha tea effect on cognitive function

**Fig. 1:**
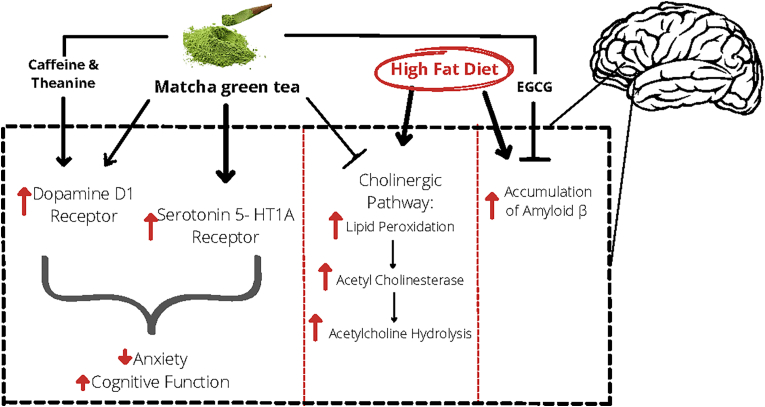
The effect of matcha and its bioactive compounds on cognitive function. Abbreviations: EGCG: Epigallocatechin gallate.

The mechanisms by which matcha tea affects cognitive function is expected to work through the action of bioactive compounds available within it (see Fig. 1). The catechin EGCG is the most active compound in matcha, which can cross the blood brain barrier (Nakagawa and Miyazawa, 1997) and protects against Amyloid β (Aβ) toxicity by inhibiting its accumulation (Gomez-Ramirez et al., 2007) and production (Yokogoshi et al., 1998). Theanine can also cross the blood brain barrier and enhance the mood (Levites et al., 2003; Bastianetto et al., 2006) by causing a favorable downshift in neurodegeneration, primarily because of its structure is analogous to glutamate, which is the main excitatory neurotransmitter in the brain (Deb et al., 2019). Caffeine coupled with theanine can also enhance cognitive function by increasing the dopaminergic and cholinergic transmissions in the brain (Sakurai et al., 2020). The antioxidant function of matcha, through the effect of EGCG, is of utmost importance because the brain structure is particularly vulnerable to oxidative stress due to its higher content of unsaturated fatty acids in contrast to other tissues (Dutheil et al., 2016). Therefore, ingestion of compounds with antioxidant activity like matcha can prevent cognitive impairment as a result of oxidative damage (Baluchnejadmojarad et al., 2009). Matcha can also protect the integrity of the antioxidant systems in the liver, brain, and blood, preventing cognitive damage (Kim et al., 2020). Another mechanism by which matcha prevents cognitive impairment is by improving the function of the cholinergic transmission system in the brain, which is strongly related to cognitive function (Kim et al., 2020). HFD consumption increases the expression of acetylcholinesterase due to lipid peroxidation, which promotes the breakdown of the neurotransmitter acetylcholine. A HFD also triggers the aggregation of Amyloid β, which induces neuronal death in the brain. However, consuming matcha showed an improvement in cholinergic function, by downregulating acetylcholinesterase, reducing the expression levels of Amyloid β and upregulating choline acyltransferase in the hippocampus, and cerebral cortex (Kim et al., 2020). The residue part of matcha was also found to activate dopamine D_1_ and serotonin 5- HT1A receptors in an animal model, resulting in inhibited or reduced anxiety (Yuki Kurauchi et al., 2019). In addition, EGCG was able to reduce neuroinflammation in the microglial cells in the hypothalamus, which lowered the risk for cognitive decline (Zhou et al., 2020).

### 3.2 Matcha tea and cardio-metabolic health

Cardio-metabolic diseases are group of disorders related to defect in glucose-insulin homeostasis such as type 2 diabetes mellites (T2DM), adiposity disorders such as obesity, and cardiovascular diseases (CVD) (Mahmood et al., 2014). Specific parameters involved in cardio-metabolic health include serum level of high-density lipoprotein (HDL) and triglycerides, blood pressure and insulin function (Cannon, 2007). Recently, consumption of matcha tea has been linked to impressive cardio-metabolic outcomes and health benefits (see Table 3). An *in vivo* study assessed the cardio-metabolic and anti-oxidative effects of consuming varied levels of matcha tea in mice fed on high-fat diet every other day for 4 weeks (Xu et al., 2016). The study explored several clinically relevant parameters such as body weight, total serum cholesterol, triglycerides, low density lipoprotein (LDL), high density lipoprotein (HDL), and blood glucose, in addition to indicators of oxidative stress such as levels of superoxide dismutase, malondialdehyde, and glutathione peroxidase. Findings point to an overall benefit from consumption of matcha tea on most parameters. Specifically, low to moderate levels (0.025–0.05%) of matcha consumption in a high-fat diet successfully prevented weight gain to levels significantly different from mice on control diet. Moderate to high levels of matcha however (0.05%–0.075%) resulted in a statistically significant reduction of total cholesterol, triglycerides, and LDL levels, as well as an increase in HDL levels. A similarly significant, albeit less intense, reduction in blood glucose levels was also reported with all levels of matcha consumption. It seems that matcha tea also contributes to an overall antioxidant benefit as mice fed on high fat-diet along with matcha demonstrated overall higher levels of superoxide dismutase and glutathione peroxidase compared to mice fed only on a high-fat diet. Interestingly, the insoluble residue of matcha tea alone were able to deliver similar results to medium and high levels of matcha, while the soluble part alone was not as effective. Therefore, since matcha residues mainly contain fibers, this supports the important role of fiber in ameliorating the deleterious effects of high fat diet (Xu et al., 2016). A more recent *in vivo* study assessed the consequences of simultaneous consumption of matcha tea in a high fat diet for 6 weeks (Zhou et al., 2021). Similarly, matcha consumption halted weight gain, and improved blood glucose levels and lipid profile in a dose-dependent manner. Additionally, it prevented accumulation of visceral and hepatic fat and maintained normal liver functions. Transcriptomic analysis also indicated upregulation of cytochrome P450 enzymes and downregulation of lipid droplet-associated proteins, further stressing on the beneficial effects of matcha supplementation on liver function (Zhou et al., 2021). Another study investigated the effect of matcha supplementation on obese mouse model found that matcha intake slowed down body weight gain, but mice were still significantly heavier than control mice at all matcha concentrations (Zhou et al., 2020). Also, matcha did not affect the appetite as food intake was higher than control for all mice on high-fat diet with or without matcha intake. Similar to previous studies, a reduction in BG was observed but not to baseline levels as the controls. In contrast, the effect on HDL was not observed, which could be explained by the longer administration period of matcha, indicating that the influence of matcha on HDL might be acute or short-lived. Nonetheless, it is clear that a total reduction in triglycerides, cholesterol, and LDL is achieved by concomitant intake of matcha in a high-fat diet (Zhou et al., 2020, 2021; Xu et al., 2016). Notably, a reduction in total cholesterol was observed in a dose-response manner as consumption of matcha increased (Zhou et al., 2020). Overall, this points that matcha has the potential to regulate blood sugar and lipids and ameliorate HFD-induced obesity.

**Table 3:** Summary of studies investigating the cardio-metabolic effects of matcha tea.

Reference	Subject	Year	Dose	Duration	Methods	Results
Zhou et al. (2021)	Male C57BL/6 mice	2021	HFD + 0.1%, 0.5% and 1% matcha vs control vs HFD	6 weeks	Tissue weight	Weight gain rate
					Body weight	food intake (kcal/d)
					Food intake	Improved BG and lipid profile
					TG, TC, LDL, Blood Glucose (BG)	Alanine transaminase, Aspartate transaminase
					Histologic analysis of epididymal adipose tissue	Upregulated cytochrome P450
					Liver tissue (lipid accumulation, ALT, AST, and inflammation: TNF-α, IL-6, IL-1β)	Downregulated lipid droplet-associated proteins
					Liver transcriptome analysis	
					PPI network analysis	
Xu et al. (2016)	Male ICR mice	2016	Control vs HFD vs HFD+0.025% or 0.05% or 0.075% matcha vs HFD+0.05% matcha extract or residue.	4 weeks	Body weight	Weight gain (best 0.05%), TC, TG, LDL, BG.
					Blood and liver samples	HDL
					Superoxide dismutase (SOD) and glutathione peroxidase (GSH-Px)	Superoxide Dismutase, Glutathione Peroxidase, Malondialdehyde (serum and liver)
					Lipid peroxidation	
Zhou et al. (2020)	Male C57BL/6 mice + Mouse microglial BV-2 cells	2020	Control vs HFD (60%E from fat) vs HFD + 0.1%, 0.5% and 1% matcha	6 weeks	Serum glucose	Weight gain
					TG, TC, LDL, HDL	BG, TG, TC, LDL, fat accumulation in epididymal area
					Adipose tissue samples	Inflammatory cytokines
					Hypothalamus samples	**X** No effect on food intake and HDL
						Inhibited JAK2 and STAT3 phosphorylation

Abbreviations: HFD, High-fat diet; IL-1β, Interleukin-1 beta; IL-6, Interleukin- 6; JAK2, Janus kinase 2; LDL, Low-density lipoprotein; PPI, Protein-protein interactions; STAT3, Signal transducer and activator of transcription 3; TC, Total cholesterol; TG, Triglycerides; TNF-α, Tumor-necrosis factor-alpha.

It is important to mention that the lipoprotein profile of rodents is different from that of humans, as HDL is the prevalent lipoprotein, possibly because rodents lack CETP in their plasma (Hunjadi et al., 2021). Therefore, although studies investigating the association between matcha tea and cardio-metabolic outcomes show promising results, more clinical studies on humans are needed to confirm these associations.

Obesity, induced by the consumption of a high-fat diet, triggers the activation of metabolic signaling pathways, which leads to an induction of low-levels of inflammatory cytokines resulting in a low-grade inflammatory response (Gregor and Hotamisligil, 2011). Major inflammatory cytokines such as tumor necrosis factor alpha (TNF-α), interleukin 1 beta (IL-1β), and interleukin 6 (IL-6) have the ability themselves to potentiate inflammation and are known to be associated with various inflammatory diseases such as hypertension, type 2 diabetes, and cardiovascular disease (Kany et al., 2019; Martin-Rodriguez et al., 2015). Ingestion of matcha was able to ameliorate this inflammatory response in obese mice, as multiple animal studies reported a suppression in the release of the major inflammatory cytokines in the blood (Wang et al., 2020; Hunjadi et al., 2021; Zhou et al., 2020). Obesity also leads to higher oxidative stress in the body characterized by excessive reactive oxygen species (ROS) that react with lipids, proteins, and nucleic acids and induce various chronic diseases. Consuming compounds with antioxidant properties is especially important to neutralize these ROS, which improves health, protects from chronic diseases, as well as aids in weight loss (Perez-Torres et al., 2021). The protective effect of matcha against oxygen radicals was found to be significantly higher than the effect of normal tea leaves due to increased catechin levels (Fujioka et al., 2016). Another analysis found that matcha has higher ability to inhibit the production of ROS compared with the same amount of loose-leaf form of green tea (Fujioka et al., 2016), all of which improves cardio-metabolic health and aids in weight loss.

#### 3.2.1 Plausible mechanisms of matcha tea effect on cardio-metabolic health

**Fig. 2:**
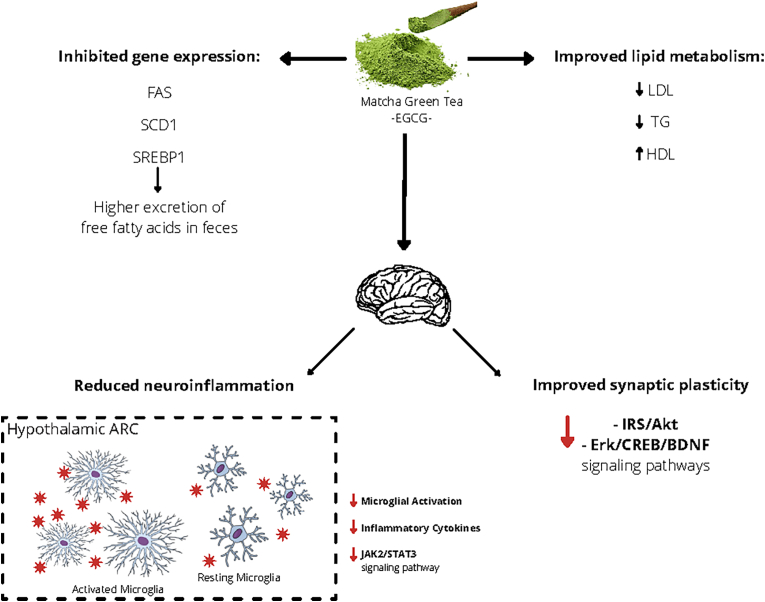
The effect of matcha and its bioactive compounds on cardiometabolic health. Abbreviations: Akt, Protein kinase B; BDNF, Brain-derived neurotrophic factor; CREB, cAMP-response element binding protein; EGCG, Epigallocatechin gallate; Erk, extracellular signal-regulated kinase; FAS, Fas Cell Surface Death Receptor; HDL, High Density Lipoprotein; IRS, Insulin Receptor Substrate; JAK2, Janus Kinase 2; LDL, Low Density Lipoprotein; SCD1, Stearoyl-CoA Desaturase-1; SREBP1, Sterol Regulatory Element-Binding Transcription Factor 1; STAT3, signal transducer and activator of transcription 3; TG, Triglycerides.

Matcha tea exhibits its cardio-metabolic health functions mainly through the power of the abundant bioactive compounds it contains (see Fig. 2). Catechins in matcha may lower the content of triglycerides and low-density-lipoprotein cholesterol in the blood and increase high-density lipoprotein cholesterol, which all leads to improved lipid metabolism and therefore, improvement in body weight and cardiac health (Bolduc et al., 2012). Specifically, EGCG, the most abundant catechin in matcha, inhibits the expression of genes involved in lipid metabolism, such as FAS, SCD1 and SREBP1, which leads to higher excretion of free fatty acids (Li et al.,2018). In addition, EGCG was shown in previous studies to improve the synaptic plasticity of the hippocampus through IRS/Akt and Erk/CREB/BDNF signaling pathways (Mi et al., 2017) and reducing neuro-inflammation by inhibiting the JAK2/STAT3 signaling pathway in the microglia cells in the hypothalamus (Zhou et al., 2020). The hypothalamus is known as the center for energy homeostasis. The microglia are the native immune cells in the CNS, which actively participate in the initiation of hypothalamic inflammation when there is a surplus of caloric intake, and they mainly work in the hypothalamic ARC. Both appetite-suppressing and appetite-stimulating neurons are found in the hypothalamic ARC, however, the ratio between excitatory and inhibitory synaptic inputs on the membranes of these neurons would be changed under different conditions of neuro-inflammation. This evidence points that matcha, through the effect of EGCG, alleviates the hypothalamic microglial hyperplasia and activation, thus reducing neuro-inflammation and protecting the energy homeostatic function of the brain that eventually controls appetite and body weight (Kim et al., 2020). In addition, *in vivo* research showed that caffeine antagonizes adenosine receptor, which leads to higher release of epinephrine and therefore, enhanced insulin sensitivity (Thong and Graham, 2002). Similarly, evidence from a recent cohort of pregnant women reported that caffeine intake reduced the risk for gestational diabetes mellitus (GDM) by decreasing blood glucose, C-reactive protein and C-peptide levels, and resulted in favorable lipid profiles (Hinkle et al., 2021). Theanine consumption has also been shown to lower blood pressure (Deka and Vita, 2011; Yoto et al., 2012) and reduce inflammation (Sergi et al., 2017) both of which relate to a lower risk for cardio-metabolic diseases. The mechanism by which theanine lowers systematic inflammation is thought to be through its involvement in the hypothalamic-pituitary-adrenal (HPA) axis responses (Kimura et al., 2007).

### 3.3 Matcha tea and anti-tumorogenesis

The anti-tumor potential of matcha is assumed to function through the multiple bioactive molecules found in it. However, to date, the synergistic effect of these molecules in whole matcha has been investigated only in three *in vitro* studies, all of which examined the effect on breast cancer cells. The study by Schröder et al. (2019) investigated the effect of matcha tea extract, EGCG and quercetin on breast cancer cells. Their results showed that matcha greet tea has similar ability to inhibit proliferation and viability of both estrogen receptor-positive and -negative breast cancer cells as its components EGCG and quercetin (Schroder et al., 2019). Another study by Bonuccelli et al. (2018) looked at the details by investigating the pathways by which matcha exerts its effects. Matcha can significantly inhibit the propagation of breast cancer cells, mainly through mTOR signaling by downregulating many components of the 40S ribosome. Their study also showed that matcha affected key pathways in the MCF7 breast cancer cells, including the antioxidant response, cell cycle regulation, as well as interleukin signaling (Bonuccelli et al., 2018). The most recent study by Keckstein et al. (2022) investigated the effect of matcha extract on the PPARγ-dependent proliferation behavior of T47D breast cancer cells. Their results revealed that a negative correlation exists between the inhibition of cell proliferation and the overexpression of PPARγ on protein and mRNA levels. Although the evidence about PPARγ in tumor cells has yielded opposing results, it is mostly reported to have a tumor suppressing effect in breast cancer cells. This suggests the possible involvement of PPARγ in the anti-tumor effect demonstrated by matcha (Keckstein et al., 2022). Overall, available evidence implies that matcha may have significant anticancer activity by intervening in the metabolic reprogramming of breast cancer cells. However, more studies are needed to examine this effect on different types of cancer cells, and there is also a need to verify it using animal models.

#### 3.3.1 Plausible mechanism of matcha tea effect on tumorogenesis

Previous literature also explored the anticancer activity of EGCG and quercetin (see Table 4). Besides steroid receptor (Hallman et al., 2017) and PPARγ receptor (Wu et al., 2017) interaction, other assumed mechanisms of action include interaction with the PI3K/Akt/mTOR signaling pathway (Ding et al., 2017), VEGF (Rashidi et al., 2017), the 67-kDa laminin receptor (67-LR) (Li et al., 2017), p53, Bax protein (Moradzadeh et al., 2017) and Bcl-2 (Huang et al., 2017). However, none of the anticancer effects of EGCG have been proven clinically. Theanine is a nonprotein derivative amino acid special to tea and is abundant in matcha. A recent article (Shojaei-Zarghani et al., 2021) systematically reviewed the recent evidence available for the anticarcinogen and anticancer effects of theanine from natural sources. The evidence from 14 *in vitro*, *ex vivo*, and *in vivo* studies concluded that theanine has moderate inhibitory effects on cancer cells’ apoptosis, metastasis, migration, and invasion, in addition to mild anti-proliferative influence on various cancer cell lines. The overall evidence from animal experiments showed that theanine exerted its anticancer function by inhibiting EGFR, VEGFR, Met, and Akt/mTOR, JAK2/STAT3, and ERK/NFκB signaling pathways, as well as activating caspase-independent programmed cell death (Shojaei-Zarghani et al., 2021). In addition, *in vivo* research showed that exposure to caffeine inhibited the development of hormone-induced breast cancer in a rat model (Petrek et al., 1985). Nonetheless, evidence from epidemiological meta-analyses showed no association between caffeine and risk of ovarian cancer (Shafiei et al., 2019), a protective effect against colon cancer but not rectal cancer (Sartini et al., 2019), and a moderately protective effect against the development of basal cell cancer, which is a type of non-melanoma skin cancer (Caini et al., 2017).

**Table 4:** Summary of studies investigating the anti-tumor effects of matcha tea.

Reference	Type	Year	Dose	Methods	Results
Keckstein et al. (2022)	In vitro	2022	5, 10 and 50 μg/ml after 72 h	Cell viability (WST-1 proliferation assay, measured as optical density)	cell viability
				PPARγ expression (PCR and Western Blot)	PPARγ expression
Bonuccelli et al. (2018)	In vitro	2018	Control vs doxycycline vs 0.2 mg/ml matcha	Ingenuity Pathway Analysis (IPA)	Mitochondrial metabolism
				Oxygen consumption rate (OCR)	Glycolysis and glycolytic capacity
				Extracellular acidification rate (ECAR)	Cell viability
				Quantitative proteomic analysis	Sphere forming ability
					Oxygen consumption rate and ATP production
Schroder et al. (2019)	In vitro	2019	Green tea or Matcha in Ethanol or water: 3000 μg/ml	MTT test for viability	Viability and Proliferation
			vs EGCG vs quercetin and tamoxifen	BrdU assay	
				ATP luminescence test/CellTiter-Glo® test	
				Neutral Red test for viability	
				Oxidative stress by measuring H2O2 concentration	

AbbreviationsATP, Adenosine triphosphate; EGCG, Epigallocatechin-3-gallate; MTT, 3-(4,5-dimethylthiazol-2-yl)-2,5-diphenyl-2H-tetrazolium bromide; PCR, polymerase chain reaction; PPARγ, Peroxisome proliferator-activated receptor gamma.

A possible limitation to all studies on matcha tea is the quality of commercial matcha tea sold in overseas markets versus in Japan. The products available in the Japanese market is of higher quality and meets more of the conditions required to produce an effect on the various health aspects, including cognitive function. The study by Unno et al. (2018) found that out of 76 product samples bought from Japan, 50 of them contained levels of theanine expected to have a stress-reducing effect (>17 mg/g), while only 6 exceeded that limit in the ones purchased from international market (Unno et al., 2018). This variation in the quality of matcha tea requires studies using it to confirm the chemical composition of the sample before evaluating its effectiveness.

## 4 Conclusion

Matcha tea started gaining popularity only recently and studies examining its effect on human health is limited. Despite the available evidence regarding its effect on cognitive function, both acutely and after long-term ingestion, the studies are heterogenous and small number in number. Also, the evidence from animal studies suggests a similar effect as shown in human RCTs while providing more molecular mechanisms. The cardio-metabolic effect of matcha was studied only in animals and is showing more coherent results indicating a significant protective effect against the metabolic imbalances caused by a high-fat diet. However, such evidence needs to be confirmed using human RCTs. The anti-tumor function of matcha has only been investigated using *in vitro* models and the results showed a significant ability to inhibit metastasis, proliferation, and viability of breast cancer cells. More research is warranted to examine the effect in other cancer cells and to be confirmed in the more complex animal models.

## Funding

Sara Sokary holds a graduate assistant grant from 10.13039/501100004252Qatar University (QUCP–CHS–2022-483). The publication fees for this manuscript is funded by Qatar National Library.

## CRediT authorship contribution statement

**Sara Sokary:** Writing – original draft, Investigation. **Maha Al-Asmakh:** Funding acquisition, Methodology, Writing – review & editing, Supervision. **Zain Zakaria:** Writing – review & editing. **Hiba Bawadi:** Conceptualization, Methodology, Writing – original draft, Supervision.

## Declaration of competing interest

The authors declare that they have no known competing financial interests or personal relationships that could have appeared to influence the work reported in this paper.

## Data Availability

No data was used for the research described in the article.
